# Supplementary material for: A New qPCR Assay for the Rapid Diagnosis of Anthonomus grandis Subspecies
Source: Insects. 2023 Oct 31;14(11):845. doi: 10.3390/insects14110845 (PMC10671495; doi:10.3390/insects14110845)
Supplement: Supplementary file 1 [file insects-14-00845-s001.zip › Supplementary Material S2 - Running and Analyzing the ANZTMGE Assay.pdf]

# Supplementary Material
# Protocol S2: Running and Analyzing the ANZTMGE Assay

## I. Introduction

### A. Scope
This assay allows for the differentiation between boll weevil (BW), *Anthonomus grandis grandis*, and thurberia weevil (TW), *Anthonomus grandis thurberiae*, using a single nucleotide polymorphic (SNP) marker.

### B. Background
This protocol uses the ANZTMGE assay developed and validated by Raszick et al. (2023). The assay is registered with Thermo Fisher Scientific as a custom TaqMan SNP Genotyping assay. It is a qPCR assay. The TW variant of the locus is fluorescently labeled with FAM, while the BW variant of the locus is labeled with VIC. The Bio-Rad CFX Maestro software can discriminate between these two species based on the fluorescence profiles of the two fluorescent dyes.

## II. Equipment and Materials

### A. Equipment
1. Dedicated pipettes, calibrated annually (0.5-2 µl, 2-20 µl, 20-200 µl, and 200-1,000 µl); optional: Multichannel pipettes, calibrated annually (0.5-2 µl, 2-20 µl)
2. Vortexer (any vendor)
3. Centrifuge for 1.5mL microcentrifuge tubes and 96 well plate
4. Freezer (-20$^o$C) (any vendor)
5. Refrigerator (4$^o$C) (any vendor)
6. Bio-Rad CFX96 or CFX384 Touch Real-time PCR thermal cycler
7. PCR workstation with positive pressure air flow (Optional)

### B. Materials and Reagents
1. 40X ANZTMGE Taqman assay (Thermo Fisher Scientific)
2. 1X TE Buffer (any vendor)
3. 2X TaqMan Genotyping Master Mix (Thermo Fisher Scientific Cat# 4371353)
4. Sterile filter pipette tips (any vendor)
5. Reagent troughs (if using multichannel pipettes)
6. Gloves (any vendor)
7. 1.5 ml microcentrifuge tubes, 5 ml microcentrifuge tubes, sterile (any vendor)
8. Tube racks for 1.5 ml microcentrifuge tubes, 5 ml microcentrifuge tubes (any vendor)
9. 96-well plates for the CFX96 real-time system, white (Bio-Rad, item#MLL9651)
    - OR 384-well plates for the CFX384 real-time system
10. Plate sealing films (ultra-clear, Bio-Rad, item#MSB1001)
11. Sealing film roller (any vendor)

### C. Reagent Preparation and Storage
1. The 40X ANZTMGE TaqMan Assay stored at -20$^o$C (see Table S1 for 20X Preparation)
2. The 1X TE Buffer stored at room temperature.
3. The 2X TaqMan Genotyping Master Mix must be stored at 4$^o$C. It cannot be re-frozen.

### D. Selection of real-time PCR controls
a. Selection of Positive Controls
    i. Include at least one positive control for TW and one positive control for BW. (The positive controls should have provided the TW or BW profile with COI sequencing information consistent with TW or BW respectively).
b. Selection of Negative Controls
    i. The PCR negative control (i.e. no template control) should be $H_2O$
    ii. The DNA isolation control (i.e., no tissue control) should be a “blank” tube reaction included during the DNA isolation of the samples selected for analysis (i.e., a DNA isolation reaction tube that included no insect tissue but was processed using standard DNA isolation protocol).

### III. Real-time PCR setup

- *Use sterilized tubes and filter tips for all steps.*
- *Use gloves for every step and change gloves if contamination is suspected.*
- *If using a 96-well plate, use the 10 µl reaction.*
- *If using a 384-well plate, use the 5 µl reaction.*

1. Defrost and dilute the 40X ANZTMGE assay. Keep assay on ice.

**Table S1**: Dilution of ANZTMGE assay to 20X

| Reagents | Volume of reagent in 5 µl reaction | Volume of reagent in 10 µl reaction |
|---|---|---|
| 1X TE Buffer | 0.125 µl | 0.250 µl |
| 40X ANZTMGE TaqMan assay | 0.125 µl | 0.250 µl |

2. Prepare a master mix as shown in Table S2. Make enough master mix for all samples (including the controls) and 8 additional samples (to account for pipette error) in a 5 ml tube. Spin down each solution prior to adding to the master mix.

**Table S2**: Reagent Volumes and Concentrations for TaqMan assay

| Reagents | Volume of reagent in 5 µl reaction | Volume of reagent in 10 µl reaction |
|---|---|---|
| 2x TaqMan Genotyping Master Mix | 2.50 µl | 5.00 µl |
| 20X ANZTMGE TaqMan assay | 0.25 µl | 0.50 µl |
| **Master Mix Volume** | **2.75 µl** | **5.50 µl** |
| **Sample Volume** | **2.25 µl** | **4.50 µl** |
| **Total Reaction Volume** | **5.00 µl** | **10.00 µl** |

3. Mix the Master Mix by inversion or gently tapping the tube with your finger.
   - If using a multichannel pipette, pour Master Mix into sterile reagent troughs.
4. Aliquot 5.5 (2.75) µl of Master Mix solution into each well of a 96-well (384-well) plate that will be processed (Note: use a multichannel pipette and reagent trough if available).
5. Add 4.5 (2.25) µl of template DNA into the appropriate well. Add 4.5 (2.25) µl of sterile $H_2O$ into the appropriate negative control well.
6. Carefully apply a sealing film to the plate using the sealing film roller. Ensure that the film is clear of smudges/scratches or particles and do not touch the film above the sample wells. (Note: You may the use the paper that is removed from the sealing film as a cover over the film on the plate while using the roller).
7. Spin down the plate for a few seconds using a plate centrifuge.
8. Raise the lid on the CFX96 (CFX384) using the button on the lid. Place the plate into the machine in the appropriate configuration (well A1 should be in the upper left). Close the lid using the button on the machine. See section IV for instructions on setting up the instrument.

### IV. PCR Run Conditions

PCR run conditions are those typical for a TaqMan SNP Genotyping Assay and do not differ from the manufacturer's recommended protocol excepting that this assay runs for 45 cycles instead of 40 (Table S3). If using a Bio-Rad CFX machine, be sure to select the "All Channels" option.

**Table S3:** Cycling Parameters

| Step | Description |
|---|---|
| 1 | 95ºC for 10:00 |
| 2 | 95ºC for 0:15 |
| 3 | 60ºC for 1:00 Plate Read |
| 4 | GOTO 2, 44x |

### V. Plate and Fluorophore setup on the CFX Manager software

1. Click <Plate Setup> on the top right corner of the CFX Manager program.
2. Select <view/edit plate…> from the dropdown menu. A new window will appear. Highlight the entire plate on the plate editor window by clicking on the top lefthand corner of the plate selection.
3. Click on <select fluorophores…> a window will appear with a list of all dyes. Five dyes are selected by default. Select the FAM and VIC fluorophores and deselect the others. Click OK.
4. Load VIC and FAM by clicking the boxes on the side of the screen. Fluorophores will be identified in each well.
5. Highlight desired wells to be excluded. These are usually empty/unused wells, but you may want to exclude cells if you know you made a pipette error. Exclude these wells by checking the box "Exclude Wells in Analysis" on the bottom right of the window.
6. To identify positive controls, select a single well and at the "Sample Type" dropdown menu, select corresponding identification of positive controls. for all controls.
7. To identify NTC and Negative controls, select the appropriate cells and identify ALL of them as NTC.
8. Optional: Indicate biological and/or technical replicates.
9. Click <OK> and click <Yes> to apply changes.

### VI. Analysis

#### A. Endpoint Analysis

1. To view the SNP assay analysis, select the Allelic discrimination tab.
2. View box "Selected Fluorophores"
   a. Make sure the right dye is labeled on the correct axis.
      i. X axis FAM
      ii. Y axis VIC
3. High light samples to be analyzed and then select box "View call map"

The results will be available using the default settings in the "Allelic Discrimination" Tab. The calls are made by the software and will display identification of individuals as homozygous for allele 1 (VIC labeled), which corresponds with BW identification, allele 2 (FAM labeled, which corresponds with TW identification, Occurrence of heterozygous for this particular assay are considered rare, so only homozygous identification results are expected. A "no call" identification will indicate no amplification for that well and should be expected for negative and non-tissue controls or can be observed in failed reactions.

#### B. Interpretation

1. If all replicates agree, the final determination will be the same as the replicated results.
2. If all replicates did not agree, the amplification curve(s) for the replicate(s) that deviated from the expected variant determination (based on collection, morphology, and/or COI data) should be examined for an exponential pattern of amplification with a plateau in later cycles. If the amplification pattern is unusual, it is likely because of equipment or reagent failure and/or human error, and the respective replicates should be discarded. If the suspects appear to indicate proper amplification, those replicates should not be discarded, and the conflicting determinations among replicates should be resolved using the following criteria.
   i. If there is a single heterozygote or "no call" determination, the other replicates should be regarded as correct and the replicate indicating otherwise should be ignored.
   ii. If there are multiple heterozygote or "no call" replicates, or if all replicates differed from one another, the final result should be "inconclusive." The complete stepwise determination process is visualized in Figure 2 of the main publication.
